# Rapid decay of perceptual memory in dyslexia

**DOI:** 10.1016/j.tics.2025.09.009

**Published:** 2026-04

**Authors:** Ayelet Gertsovski, Merav Ahissar

**Affiliations:** 1 The Edmond and Lily Safra Center for Brain Sciences, The Hebrew University of Jerusalem, Jerusalem 9190401, Israel

**Keywords:** perceptual memory, categories, adaptation, statistical learning

## Abstract

The basic mechanisms that underlie developmental dyslexia – a difficulty in acquiring reading expertise – are still debated. We propose that such difficulties should be understood within the broad framework of learning and skill acquisition. Behavioral and neural studies, as well as computational analyses, imply that acquiring expertise has atypical dynamics in dyslexia, largely due to reduced perceptual memory, which is manifested in faster decay of perceptual traces of both speech and non-speech stimuli. This faster behavioral decay is associated with faster decay of neural adaptation to stimulus regularities in perceptual cortices. We propose that these atypical dynamics lead to a slower accumulation of language statistics, manifested in reduced complexity of perceptual categories, slower acquisition of words, and – counterintuitively – larger relative difficulties as exposure to stimuli grows.

## Highlights

Most people with dyslexia have reduced memory for the perceptual characteristics of stimuli (mainly auditory) to which they have been exposed.
Perceptual memory for both speech and non-speech sounds is reduced in dyslexia.
This reduction is correlated with faster decay of stimulus traces in perceptual cortices.
Reduced perceptual memory impedes the acquisition of complex perceptual categories and language-specific statistics.

## What is the cognitive basis of dyslexia?

Written script is a relatively recent invention: the first Mesopotamian proto-cuneiform script originated ~5000 years ago [1] and the alphabet originated <4000 years ago with Semitic writing [2]. Reading is thus not acquired automatically with maturation – and indeed, until the 19th century, only a small fraction of the population was literate. Instead, reading requires explicit practice to reach proficiency, like many other perceptual and cognitive skills. Basic decoding of the alphabet is typically gained within months (although rates differ between orthographies [3]). However, fast and fluent command of written script requires massive exposure. Most readers improve their reading fluency well into high school, which amounts to ~10 000 h of reading practice (~3 h per day for ~365 days a year over 10 years), similar to the acquisition rate of other complex skills [4]. The development of reading expertise involves learning to quickly retrieve the information necessary to identify the components of words, such as syllabic structure and morphology, which are largely language-specific [5].

However, practice is not always sufficient. About 5–10% of the population [6] do not become expert readers despite extensive practice. Individuals whose reading difficulties are substantially greater than expected given their general non-verbal cognitive skills are diagnosed with dyslexia [7]. We propose here that limited verbal memory, which is known to characterize dyslexia, is a core impediment to implicit learning of language-specific statistics, and consequently to the acquisition of reading expertise. We further propose that reduced verbal memory in dyslexia is one manifestation of a broader reduction in **perceptual memory** (see Glossary), particularly auditory memory.

**Figure 1:**
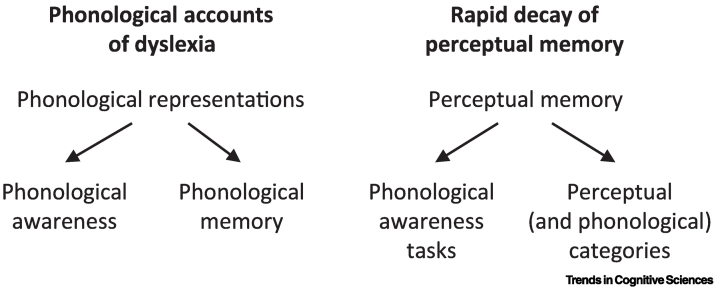
Comparison of rapid decay of perceptual memory (anchor) versus phonological accounts. Phonological accounts of dyslexia (e.g., [8]) suggest a core deficit at the level of phonological representations, which leads to difficulties in other processes such as phonological awareness and phonological memory. Our account suggests that a main deficit in dyslexia is rapid decay of perceptual memory. This memory deficit affects performance in phonological awareness tasks which often rely on memory. Importantly, it also leads to reduced accumulation of stimulus statistics, which leads to lower-resolution perceptual (including phonological) categories.

This proposal contrasts with traditional theories of dyslexia that attribute reading and additional difficulties, including atypical memory, to a core phonological deficit [8,9] (Figure 1). Proponents of these theories have primarily focused on speech-based tasks, in line with classic models that emphasize the phonological aspect of short-term memory [10]. However, we argue that individuals with dyslexia also show atypical perceptual memory in non-speech tasks. Thus, the traditional perspective cannot fully account for the range of difficulties observed in dyslexia. Advancing from our earlier proposal, where we discussed a memory difficulty in dyslexia (an **anchoring** deficit [11]), we further propose: (i) an underlying neural mechanism – rapid decay of perceptual traces (anchors) in the cortex, resulting from faster decay of stimulus-specific neural **adaptation**, and (ii) long-term consequences – slower acquisition of language statistics including **perceptual categories**.

## Limitations in perceptual memory rather than phonological skills

Individuals with dyslexia have difficulties in perceptually segmenting words into their basic sound units, known as phonemes. The ability to recognize that words are composed of these small units – referred to as phonological awareness – is fundamental to the alphabetical system which maps written symbols (orthography) to basic sound units (phonology) and vice versa. For many years reduced acquisition of reading skills was attributed to poor phonological representations or difficulties in accessing these representations [8]. Indeed, individuals with dyslexia perform worse in tasks designed to assess phonological awareness compared to typical readers [12]. However, we propose that previous studies have placed disproportionate attention on phonology as the core deficit.

The statistical power of phonological skills in capturing the unique difficulties that characterize dyslexia has been challenged based on two lines of study. First, phonological skills alone do not provide a strong classifier for dyslexia (e.g., [13., 14., 15., 16.]). Second, dyslexia is characterized by a range of non-phonological difficulties in perceptual and attentional tasks [11,17., 18., 19.]. We now further question whether phonological deficits are the primary cause of reading difficulties. Phonological awareness needs to be trained at the onset of reading acquisition, and slow acquisition of phonological awareness, which could stem from reduced perceptual memory, may lead to slow initial acquisition of reading. Because reading experience itself improves phonological processing, it follows that individuals with dyslexia – who by definition have impaired reading skills – also tend to exhibit poorer phonological skills [20,21]. Moreover, there is no strict one-to-one correspondence between tasks and cognitive constructs. Performance in phonological awareness tasks is often limited by phonological (perceptual) memory, namely the ability to retain active access to several speech components while manipulating them. For example, children with dyslexia are worse at detecting an oddball word that does not share a common sound with other words [22]. Although this was originally interpreted in the context of poor phonological skills, it may instead reflect the cognitive demands of holding and manipulating speech sounds in memory. Indeed, it has been observed that the phonological tasks that pose difficulties to individuals with dyslexia are tasks that rely on short-term memory [23].

Dyslexia is largely heritable, but its genetic basis is complex and polygenic [24]. The genetic risks of dyslexia overlap with those of many other neurodevelopmental disorders that are not related to phonology, such as dyscalculia [25]. This genetic overlap suggests that a common heritable factor, such as perceptual memory rather than phonological awareness, may underlie these conditions. This is supported by the observation that the non-word repetition span of parents is the best predictor of reading difficulties in their children [26] and is a marker of a language disorder [27].

Thus, our main claim is that individuals with dyslexia have reduced perceptual memory. Poor perceptual memory impedes their acquisition of reading through its impact on reading-related skills, including but not limited to phonological awareness. In line with recent suggestions that multiple deficits, rather than a single core deficit, are necessary to explain dyslexia [13., 14., 15.,^28^,^29^], we do not claim that reduced perceptual memory is the single cause for dyslexia. However, we do propose that it is a core deficit that is prevalent, has a genetic basis, and explains both phonological and non-phonological difficulties. Surprisingly, perceptual memory has rarely been addressed in the studies of dyslexia, perhaps because it was not considered to be important for long-term skill acquisition. Classic cognitive models of memory hypothesized that stimulus-specific memory decays within hundreds of milliseconds, and longer forms of memory gradually lose stimulus specificity [30]. However, many subsequent studies showed that memory of specific items retains perceptual specificity. This long-term specificity applies to both linguistic items such as number words [31] and to visual stimuli [32].

## Reduced perceptual memory in dyslexia impedes perceptual performance

Several laboratories have investigated whether auditory perceptual skills are atypical in dyslexia, focusing on performance in **two-tone pitch discrimination**. In this task, participants are asked to determine which of two serially presented pure tones has a higher pitch. Many variations of this task have been administered to people with dyslexia at various ages, and consistently, they show lower performance compared to cognitively matched typical readers (e.g., [33., 34., 35., 36.]; meta-analysis in [37]). Because auditory tasks require comparison of sequentially presented stimuli, poor performance may stem from difficulty in retaining a reliable memory trace of the first stimulus until the second is presented. However, successful performance depends on more than only within-trial working memory. Answers provided by participants are also strongly influenced by the statistics of sounds in previous trials, whose optimal magnitude can be quantified within a **Bayesian framework** [38]. These **perceptual biases** can be substantial, and their impact depends on how effectively participants learn the statistical structure of the stimuli throughout the experiment.

Almost 20 years ago our laboratory administered two different two-tone pitch discrimination protocols to teenagers with and without reading difficulties. In one protocol, a repeated reference tone (1000 Hz) was presented in every trial. The frequency of the non-reference tone was higher. In the second protocol, no reference tone was included [39]. Teenagers without reading difficulties showed much better performance with the structured regularity of the reference tone, which indicates a large cross-trial effect of implicit perceptual memory. To our surprise, the performance of teenagers with dyslexia was not improved by this regularity.

A similar deficit was found in speech identification tasks: participants with dyslexia did not benefit from the repetition of a small set of words across trials. We termed this automatic, implicit perceptual memory an 'anchor' and proposed that individuals with dyslexia have a reduced anchor [11]. Group differences were also found in a serial visual discrimination task [40] and an amplitude rise time task [41], both of which used reference stimuli. Importantly, reduced benefit from sound regularities is not a general characteristic of developmental disorders. For example, it does not characterize individuals with attention-deficit hyperactivity disorder (ADHD) whose reading skills are typical [42].

Reduced benefits from simple statistical regularities were also observed in other tasks, for example in a task where the participant is asked to identify within-noise repetition (a 1 s noise stimulus composed of a repeated 500 ms segment) [43]. People can learn to detect previously encountered repetitions in this task [44], and individuals with dyslexia also succeed in this challenging detection [45]. However, they are less sensitive to the cross-trial reoccurrence of noise, and tend to be more sensitive to repetitions in novel noise stimuli [43]. Nevertheless, despite a consistent trend, the group (dyslexia versus typical readers) × condition (with and without regularity) interaction was not always statistically significant [46,47], perhaps because such an interaction is based on comparing two noisy assessments (with and without regularities). A meta-analysis of eight studies concluded that there is an overall moderate, statistically significant effect of reduced anchoring in dyslexia [48].

## Weighting of perceptual priors in dyslexia is lower than expected

**Figure 2:**
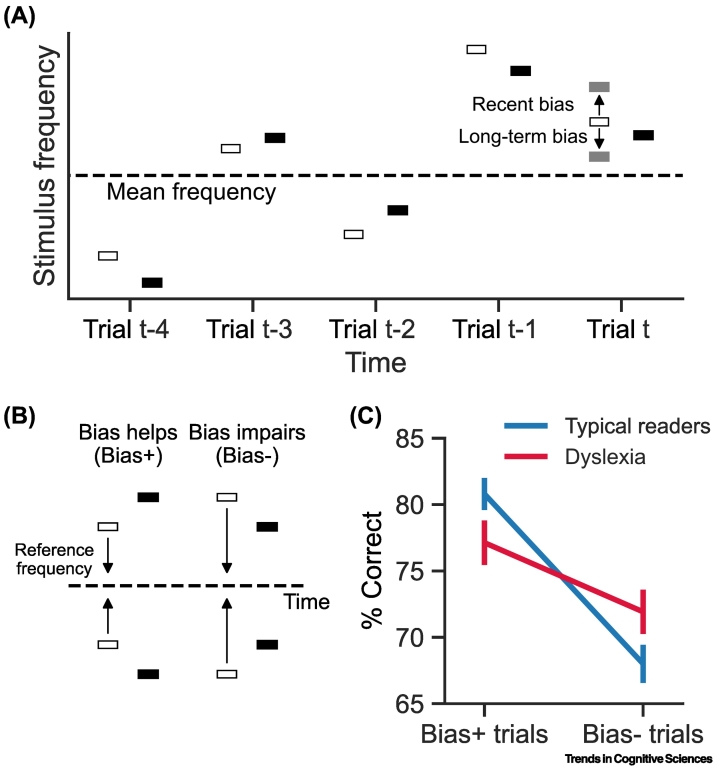
The impact of previous trials on perceptual performance is reduced in dyslexia. In serial discrimination, the first stimulus (s1, white bars) needs to be kept in active memory until the second stimulus (s2, black bars) appears, which yields a noisier representation. Bayesian reasoning asserts that this extra noise should lead to integration of priors into the representation of s1. (A) Illustration of two types of biases that integrating priors introduces on s1 in trial t: the most recent trial (t − 1) pulls toward its stimuli, whereas the longer-term bias pulls toward the mean frequency of all previous stimuli. In practice, these biases mostly operate in the same direction (when stimuli are distributed uniformly or normally), and are thus difficult to dissociate using this simple method. The biased representation of s1 in trial t is denoted by gray bars. (B) Bias^+^ versus bias^−^ trials: when s1 (white bars) is closer to the prior than s2 (black bars), the bias effectively increases the distance between the representations of the two stimuli and improves performance (Bias^+^ trials), whereas the opposite effect occurs when s2 is closer to the prior (bias^−^ trials). The accuracy difference between the two conditions is a simple measure of the bias. (C) Bias is smaller in dyslexia: bias was larger in typical readers (blue) than in dyslexia (red) in a visual serial spatial frequency discrimination task. Panel adapted, with permission, from [51]. Error bars denote the standard error of the mean. Note that reduced sensitivity to previous statistics improves performance when these statistics are misleading (bias^−^ trials).

One way to reduce the noise introduced by comparing two noisy assessments of pitch discrimination is to analyze the impact of regularities within a single protocol. This analytic advance was made possible by using computational tools to quantify how previous stimuli contribute to the formation of perceptual priors which affect perceptual decisions. Briefly, to assess the impact of simple statistics, we can compare performance between trials expected to benefit from stimulus history and those expected to be disadvantaged by it (Figure 2). Such comparisons consistently show that people with dyslexia are less affected by the history of stimuli in the tone discrimination task [49,50].

Similar behavioral results were found in a homologous visual serial discrimination task in which participants were asked to judge which of two serially presented gratings had denser stripes [51]. When both visual stimuli are presented simultaneously, the two groups perform similarly [40]. This is consistent with findings that visuospatial resolution remains intact in dyslexia when stimuli can be kept within a single memory frame (e.g., [52,53]). Interestingly, performance in the serial spatial frequency discrimination tasks does not correlate with the parallel version even though these tasks are nearly identical. By contrast, performance in the serial task correlates with verbal memory spans, which suggests a perceptual memory bottleneck that is common to visual and verbal perceptual memory [54].

## Rapid decay of perceptual memory: behavioral and neural evidence

**Figure 3:**
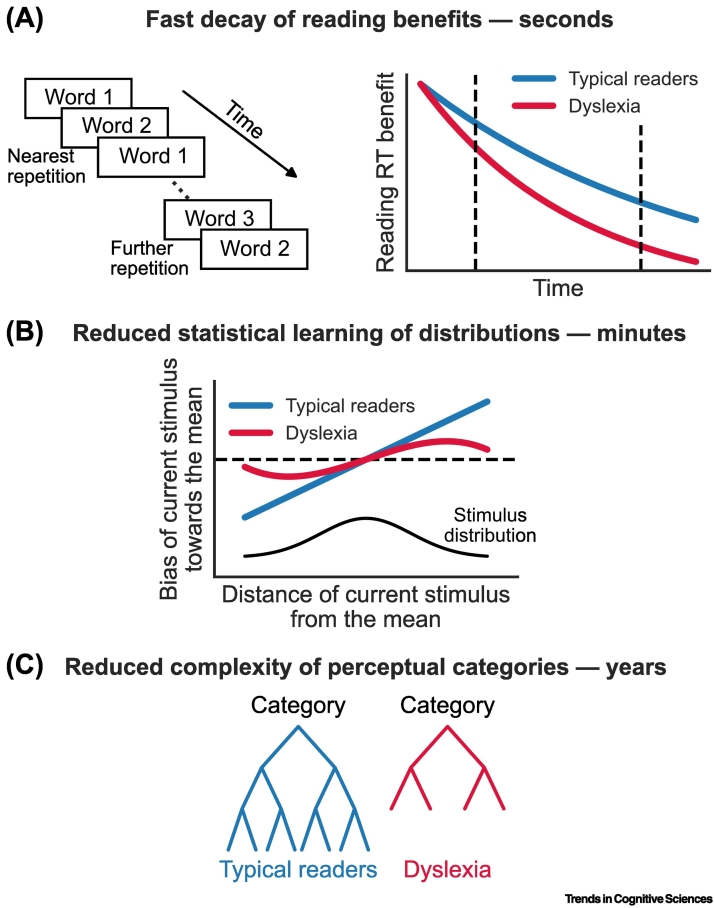
Effects of reduced perceptual memory at different timescales. (A) The reading rate benefits of recent exposure (within seconds) to the same non-word decay faster in dyslexia. (Left) Participants read aloud non-words which were repeated with one to many intervening non-words. (Right) The expected and observed results, schematically illustrated. Participants with (red) and without dyslexia (blue) respond faster to the second presentation of the same non-word. This memory-based response time (RT) benefit is expected to decrease faster in dyslexia (solid lines). This was measured for short (<2 s) and longer (>2 s) inter-repetition intervals (vertical dashed lines). Panel adapted, with permission, from [50]. (B) Reduced learning of stimulus distribution (across minutes) in a two-tone pitch discrimination task. Panel adapted, with permission, from [65]. Bias is quantified as the tendency of participants to perceive (or remember) the first stimulus in each trial closer to the mean of all stimuli. In a Gaussian distribution (indicated with a black curve), optimal bias produces a linear function which reflects the rate of decay of the probability of a stimulus as distance from the mean increases. The bias function of typical readers is optimal, which indicates implicit learning of frequency distribution (blue). In dyslexia (red), the bias is smaller and is not linear, which reflects reduced learning of the distribution. (C) A schematic illustration of the hierarchical representation of trained categories in typical readers (blue) and dyslexia (red). We suggest that categories are acquired by implicit learning of distributions. Hence, across years, reduced learning of distributions in dyslexia leads to the formation of lower-resolution categories, with shallower hierarchies.

Memories of recent events decay as more time passes. The rate of decay of memory traces in people with and without dyslexia was studied using a simple pitch discrimination paradigm in which four different inter-trial intervals (ranging from 1.5 to 9 s) were tested in separate blocks [50]. For all participants, the behavioral effect of previous trials on perceptual decisions decays with increasing inter-trial intervals. But it decays significantly faster in dyslexia. A similar pattern was found in non-word reading, where recent exposure to the same non-word facilitates reading rate (Figure 3A). As with pitch discrimination, the benefit in reading rate decreases when the interval since previous exposure is increased, and it decreases more rapidly in dyslexia [50]. Similarly, the influence of previously experienced information diminishes more rapidly in dyslexia in a visual reinforcement learning task [55]. Together, these results suggest that perceptual memory decays faster in dyslexia.

Several imaging studies suggest that atypical cortical adaptation may underlie the faster decay of perceptual memory in dyslexia. Reduced (and faster decay of) adaptation in dyslexia has been observed for a broad range of stimuli in stimulus-specific cortical areas, such as for speech sounds in perisylvian areas and for faces in the fusiform face area [56]. Faster decay of adaptation was found in the two-tone pitch discrimination task discussed above, as measured by early (100 and 200 ms delay) event-related potential (ERP) responses originating from the auditory cortex [50]. In a follow-up fMRI study using a similar paradigm with different inter-trial intervals, a tendency for faster decay of adaptation in dyslexia was observed in all regions activated by the auditory stimuli, including a significant effect in the primary auditory cortex [57]. In a subsequent fMRI study, where two-tone pitch discrimination was administered with and without a cross-trial reference tone, the auditory cortex of typical readers showed greater adaptation in the reference protocol and an accompanying behavioral improvement. No such behavioral improvement and stimulus-specific neural adaptation were found in the dyslexia group [58]. In line with these observations, a reduced sensitivity of early auditory ERP components to a syllable pitch reference was also found in children with dyslexia [59]. At the anatomical level, geometrical characteristics (gyrification) of the superior temporal gyrus (where the auditory cortex is located) differed between individuals with and without dyslexia [60] and were correlated with performance in an amplitude rise time discrimination task. This task, which was administered with a repeated reference, was more challenging for children with dyslexia [41].

Importantly, reduced adaptation in all these studies was observed by fMRI in perceptual brain areas, and in ERP in early response components attributed to perceptual areas. This atypical processing within early regions of the perceptual pathways, which is associated with memory, further supports the hypothesis that implicit perceptual memory decays more rapidly in dyslexia. Some evidence also suggests that neural atypicalities are present in subcortical regions associated with processing of auditory and visual stimuli [61., 62., 63.].

## Reduced accumulation of stimulus statistics leads to lower-resolution perceptual categories

Perceptual decisions are affected by both recent stimuli (~<5 s) and stimuli encountered further in the past. Accumulative statistics characterize long-term regularities of language – such as those related to syllables and morphology – whereas recent statistics integrate context effects [64].

To assess the impact of each timescale, two-tone pitch discrimination was used to separately assess the influence of the frequency of the most recent trial and the accumulative statistics in the experiment [65]. These analyses suggest that the influence of the most recent trial on perceptual decisions follows a 'tuning curve', in which the maximum effect occurs when the frequency difference is about half an octave. People with dyslexia show a similar shape and magnitude for recent effects, which suggests a similar use of very recent statistics. However, the effect of accumulative statistics is noisier in dyslexia. Unlike typical readers, whose perceptual decisions make optimal use of the overall frequency distribution in the experiment (similarly to an **ideal observer**), individuals with dyslexia show reduced benefits and a decision pattern indicative of suboptimal frequency distribution learning (Figure 3B). A reduced benefit from distributional learning was also observed when speech stimuli were used [66], and a larger reliance on recent over accumulative information in dyslexia was also found in learning visual–phonological associations [67].

These observations are in line with a faster decay of perceptual memory in dyslexia. They suggest that this decay impedes adequate learning of longer-term statistics, potentially reducing the richness and reliability of learned perceptual categorical prototypes in dyslexia (Figure 3C). Reduced richness, namely reduced complexity, might be manifested as either over-generalization (e.g., across contrasting syllables) or under-generalization (discrimination between stimuli belonging to the same phonological category [68]). Perceptual categories, including but not limited to phonological categories, develop over years of experience [69] and are continuously enriched by integrating stimulus statistics throughout life.

Are the difficulties in forming categories specific to auditory stimuli? Most evidence for reduced anchoring and impoverished category formation in dyslexia comes from the auditory and linguistic domains, which have also been the primary focus of research to date (e.g., [70., 71., 72., 73., 74.]; reviewed in [75]), and there is evidence for greater difficulties in dyslexia in the auditory compared to the visuomotor modality [76]. However, there are also reports of atypical visual categories (e.g., [51,77]).

To directly test visual category learning in dyslexia, a face categorization task was administered using morphs of two unfamiliar faces [78]. Although no feedback was given, categorization of typical readers improved with practice, whereas the learning slope was significantly shallower in dyslexia. These results suggest that atypical categorical learning in dyslexia is not specific to auditory stimuli, although the extent of domain-generality remains unclear. Importantly, unlike with sounds or speech stimuli – where reduced exposure to reading could potentially explain learning difficulties – there is no reason to suspect that individuals with dyslexia have less experience with faces. Thus, difficulties in face category learning cannot be attributed to reduced exposure. These findings point to a core deficit in forming categories. However, this deficit likely does not characterize all people with dyslexia, suggesting that atypical categorization may arise from multiple underlying mechanisms.

Why does a general memory deficit result in a seemingly specific difficulty in acquiring reading expertise? Reading is a unique skill because it is systematically assessed as part of formal education. Consequently, difficulties with reading can directly affect educational achievements, often making early diagnosis a priority. By contrast, potential difficulties in acquiring expertise in face recognition, for example, may not be diagnosed at all. This is evidenced by the fact that research on congenital prosopagnosia, an innate difficulty in acquiring expertise in face recognition, began only ~25 years ago [79]), whereas dyslexia has been studied for over a century [80]. Furthermore, although there is evidence for difficulties in visual memory in dyslexia, they were less systematically assessed and are perhaps less prevalent than auditory-speech memory difficulties.

## Lower-resolution categories are manifested in reduced spans

**Figure 4:**
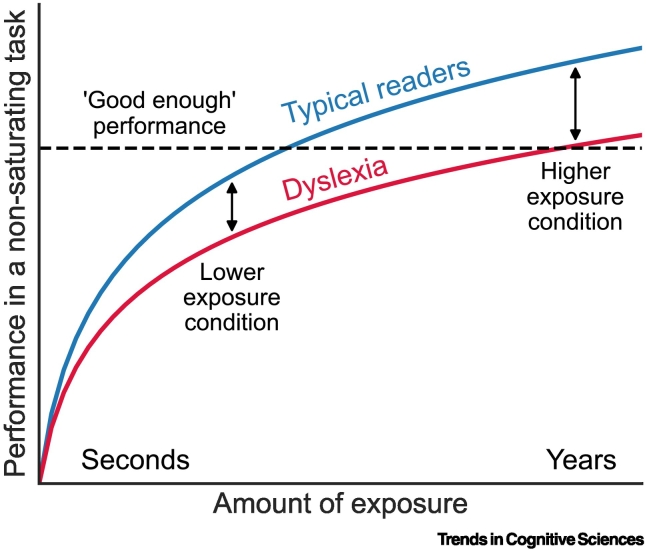
In tasks benefiting from perceptual memory, group differences increase with exposure. A theoretical predication: tasks such as span tasks, where performance improves as a function of exposure, with no saturation, will show an increase in group difference. Although both groups will benefit from exposure, individuals with dyslexia (red) are expected to have a shallower slope than typical readers (blue). This appears to be counterintuitive because typical measures of performance, such as accuracy, reach saturation, which results in both groups achieving 'good enough' performance (100% correct for easy cases) with sufficient exposure, which can be misinterpreted as 'catching up'. Figure adapted, with permission, from [109].

A reduced (noisier) rate of accumulation of long-term statistics predicts that people with dyslexia will not 'catch up' with training. Instead, group differences will increase with continued exposure. This may seem counterintuitive because people with dyslexia do learn with experience and typically attain 'good enough' performance in reading and related tasks. However, learning itself is a non-saturating process. It improves indefinitely with practice, although improvement per exposure decreases, as is evident for reaction time [81,82]. What saturates are measures of improvement such as accuracy, which can reach 100% and cannot measure further improvement. Hence, when assessed with non-saturating measures, group differences are expected to increase with equal amounts of practice (Figure 4).

Language is an important example of repeated exposures to relatively stable statistics; for instance, individuals continually encounter native language speech syllables whose statistical properties are refined and updated throughout life. The straightforward but non-intuitive prediction that group differences will increase with similar amounts of practice was tested using syllable **span tasks** with both frequent and infrequent syllables [83]. Although Hebrew-speaking participants with and without dyslexia both demonstrated larger spans for frequent compared to infrequent syllables, the benefit of greater long-term exposure to frequent syllables was significantly larger in typical readers.

This prediction was further tested using a digit span task. Digit span is particularly relevant because low scores in this task are one of the major characteristics of dyslexia [84,85]. Digit spans in the first language and the (highly-practiced) second language (Hebrew and English, respectively) of participants were compared. Although both groups manifested the expected tendency for a higher memory capacity in their first language because of greater exposure, people without dyslexia had a highly significant difference in their span scores. Crucially, the first language advantage was significantly larger in this group of typical readers than in dyslexia, consistent with the hypothesis that typical readers derive greater benefits from similar amounts of exposure [86].

Larger group differences with greater experience were also demonstrated in a task that directly mimics learning to read (learning associations between symbols and artificial speech sounds) [87]. In addition, some studies show that the sensitivity to statistical regularities that characterize language, such as phonological regularities [88,89] and morphology [90,91], is less effective in dyslexia, at least under some conditions [92]. This may be another manifestation of a general difficulty in learning auditory statistical regularities which results in group differences even for highly familiar stimuli.

Importantly, although more effective perceptual memory can support broad skills such as reading and can improve performance on specific experimental tasks, it is not always beneficial. Reduced reliance on past perceptual details in dyslexia may lead to a greater focus on content over form, and could guide individuals with dyslexia toward fields such as arts [93] and entrepreneurship [94] which may emphasize novelty more than fine verbal memory. This aligns with reports of increased creativity in adult individuals with dyslexia [95].

## Distributional versus conditional statistics

As discussed earlier, we propose that a perceptual memory deficit in dyslexia hampers the ability to accurately accumulate complex and detailed stimuli distributions. Importantly, the measures we have described denote learning of zero-order (distributional) statistics that are represented in perceptual cortices. This differs from first-order statistics (conditional probability) which may be primarily represented elsewhere in the brain (e.g., in the hippocampus [96]).

We note this distinction (theory reviewed in [97]) because the term 'statistical learning' in the psychology literature often refers exclusively to first-order statistics. This confusion between theoretical concepts and experimental protocols may stem from the title of the seminal work by Saffran and colleagues, 'Statistical learning by 8-month-old infants' [98], which demonstrated that infants exposed to a continuous syllable stream were sensitive to high transitional probabilities between syllables that make up 'words' versus low transitional probabilities across 'words'.

Many subsequent studies have used this protocol to characterize learning of conditional probabilities, both in the general population (reviewed in [99]) and in dyslexia. Although task performance is usually above chance level, it is often low even in the general population (e.g., [100., 101., 102.]), which makes it difficult to reliably evaluate whether there are true group differences. Indeed, the findings have been mixed, and some studies report significant group differences [101., 102., 103.] whereas others find no such differences [104]. Importantly, studies that measured memory for novel items (zero-order statistics) separately from first-order statistical learning tasks found significantly reduced performance in individuals with dyslexia [105].

A more direct protocol for assessing learning of transitional probabilities is the Hebb learning task [106], where recall of sequences of items presented in a fixed order (usually syllables) is compared to recall of sequences composed of the same basic units, but where the units are presented in a random order. The Hebb protocol has been administered to individuals with dyslexia but has given mixed results (impaired [107] or intact [108] learning). To dissociate between the impact of zero- and first-order statistics, this protocol was administered twice, once with frequent and once with infrequent syllables. Comparison of mean performance between frequent and infrequent syllables was used to assess sensitivity to long-term probabilities, whereas the Hebb repetition effect measured sensitivity to conditional probabilities [109]. Importantly, the Hebb repetition effect was intact in dyslexia. However, overall scores were higher in typical readers when high-frequency syllables were used, which suggests that they are able to gain more from long-term, zero-order language statistics compared to individuals with dyslexia.

Another task that measures first-order statistics, but in a sensory-motor context, is serial reaction time (SRT), whereby participants who are presented with a fixed sensory-motor sequence become faster specifically with the repeated sequence. SRT was also assessed with dyslexia, but also with mixed results (group differences [110., 111., 112.] versus null findings [104,113,114]).

Overall, it is not clear whether these different tasks, which all aim to measure first-order statistics under different protocols, are based on shared neural mechanisms [115]. Any generalization will require better understanding of these different manifestations of statistical learning.

## Concluding remarks

Reading is a complex task, and reading difficulties have complex genetics. Hence, a single core deficit is unlikely to explain all manifestations in all individuals with dyslexia. However, we suggest that reduced perceptual memory may be a central deficit that accounts for both phonological and non-phonological difficulties. In terms of behavior, there is evidence that perceptual memory decays more rapidly in dyslexia. At the neural level, this is reflected in perceptual cortices as atypical, faster decay of stimulus-specific adaptation. The role of perceptual memory in forming long-term representations has been understudied even in the general population, perhaps because it is conceptually situated between the traditionally separated cognitive constructs of perception and memory. Analysis of perception from a Bayesian perspective allows researchers to quantify the optimal contribution of memory to task performance. Calculation of perceptual priors and their weight in perceptual decisions indicates that this weight is lower in dyslexia compared to optimal use. However, much work remains to be done to better understand perceptual memory across stimuli and modalities, and its relation to reading difficulties (see Outstanding questions).

Outstanding questions Why do some people with reduced perceptual memory develop dyslexia whereas others either have typical learning dynamics or mainly have other atypicalities?

How does reduced perceptual memory affect the development of abstract cognitive skills? Does it stimulate in-depth analyses of stimuli in dyslexia such that retention relies on more abstract forms of memory?

Are perceptual memories for different modalities and different stimuli aspects (e.g. faces versus spatial relations) correlated?

What is the common genetic factor in perceptual memory?

What are the neural substrates of zero-order statistics versus first-order statistics? Are they separated (e.g., cortical for objects and hippocampus for serial order)?

Alt-text: Outstanding questions

## Glossary

**Adaptation**: in the context of cognitive neuroscience, adaptation is characterized by a reduction in neuronal activation (or in an imaging-based measure of activation) induced by the repeated presentation of a stimulus compared to activation induced by its previous presentation.
**Anchoring**: the gradual formation of an internal representation of a stimulus with its repeated presentation.
**Bayesian framework**: this framework of perception assumes that perception is the outcome of combining prior knowledge of the statistics of stimuli with current sensory data. The relative weight of each source is determined by its estimated reliability.
**Ideal observer**: a hypothetical performer whose perceptual priors and their weighting in perceptual decisions are optimal given the sensory noise that the modeled participant has.
**Perceptual bias**: a phenomenon where perceptual decisions are implicitly affected by previous stimuli. We refer here to contraction bias – where choices are based on stimulus representations that are pulled towards representations of previous stimuli, both recent stimuli and across longer intervals. The bias can be quantified based on the series of decisions made by the participant in perceptual tasks.
**Perceptual categories**: a representation of the dimensions or qualities that are shared by a set of similar but not identical events. Such representations are formed automatically and represent the grouping of accumulative stimuli statistics.
**Perceptual memory**: implicit and automatic memory of stimuli (visual, auditory, or other) which is based on their perceptual characteristics and does not require explicit intention.
**Span task**: a commonly used task aimed to assess short-term memory capacity. Participants are asked to repeat a series of items (e.g., syllables, digits, non-words) which is increased in size with successful performance. The number of successfully repeated items is used to measure performance.
**Two-tone pitch discrimination**: an auditory discrimination task. Two tones are presented serially and participants are asked to determine whether the first or the second tone had a higher pitch. A common protocol includes a fixed reference tone (e.g., always in the same position or always the lower tone). Perceptual bias is typically calculated in protocols where there is no reference tone.
